# Electrochemistry Cracks the P–O Bond: Sustainable Reduction of Phosphates to Phosphorus

**DOI:** 10.1021/acscentsci.3c00056

**Published:** 2023-02-21

**Authors:** Eva M. Nichols

**Affiliations:** Department of Chemistry, University of British Columbia, Vancouver, British Columbia V6T 1Z4, Canada

Elemental white phosphorus (P_4_), a precursor for many phosphorus-containing commodity chemicals, is conventionally produced by thermal reduction of phosphate ores in an energy-intensive electrical furnace. In this issue of *ACS Central Science*, Yogesh Surendranath and co-workers establish electrochemical conditions for the activation of strong P–O bonds and report the alternative synthesis of P_4_ via electroreduction of phosphate salts in molten electrolytes.^1^

Phosphorus plays a critical role in biology (e.g., in the backbone of ribonucleic acids DNA and RNA, and the triphosphate of ATP used in cellular energy transfer), agriculture (e.g., as phosphate fertilizers or the herbicide glyphosate), and the chemical industry (e.g., as detergents, pharmaceuticals, and flame retardants). The principal method for extraction of industrially relevant phosphorus is the “wet process”, where phosphate-containing ores are converted to phosphoric acid.^2^ Lower-valent phosphorus compounds are instead produced via the intermediacy of P_4_.^2^ This pyrophoric and phosphorescent solid was first isolated by alchemists in the Middle Ages, who obtained it upon distillation of urine from sand and carbon.^3^ Interestingly, this early procedure bears striking resemblance to the modern “thermal (Wöhler) process”, wherein P_4_ is produced by arc furnace treatment of phosphate ores in the presence of carbon coke (C) and silica (SiO_2_). Here, the carbon acts as a reductant and the silica as an oxide scavenger. The Wöhler process requires temperatures of up to 1500 °C and consumes between 12 and 15 MW h of electricity per ton of P_4_ produced.^3^

The low energetic efficiency and significant carbon footprint of conventional P_4_ production have motivated numerous efforts to improve sustainability of organophosphorus manufacture. For instance, Cummins and co-workers showed that several phosphorus fine chemicals can be accessed by treatment of phosphoric acid with trichlorosilane, a process that avoids the use of P_4_ as a precursor.^4^ Several reviews and perspectives have since highlighted emerging methods for the synthesis of organophosphorus compounds starting from greener inputs.^5,6^ A complementary effort involves exploring the possibility that phosphate reduction to elemental phosphorus could be driven using sustainable electricity. Building on a recent proof-of-concept report,^7^ Surendranath and co-workers have now developed the tools and conceptual framework necessary to enable efficient and selective electrochemical P_4_ generation from phosphate melts.

The role of SiO_2_ in the traditional Wöhler process can be understood in the context of Lux–Flood acid–base theory, where SiO_2_ is a Lux acid (oxide acceptor) and the phosphate starting material is a Lux base (oxide donor). The present authors hypothesized that increasing the Lux acidity of the electrolyte could facilitate reductive cleavage of strong P–O bonds. This is analogous to more familiar electrochemical reactions such as O_2_ or CO_2_ reduction that are promoted in Brønsted–Lowry or Lewis acidic media. They furthermore proposed that addition of oligomeric phosphates containing phosphoryl anhydrides (P–O–P linkages) could increase the Lux acidity of the molten electrolyte in a tunable fashion.

Testing of these ideas required construction of a specialized electrochemical cell capable of operation at 800 °C, the temperature used to achieve a molten electrolyte (Figure 1A). Carbon was found to be suitably inert under these forcing conditions; consequently, graphite rods were used as the working, counter, and pseudoreference electrodes (calibrated vs a Na/Na^+^ reference) whereas the bottom of the cell was composed of a glassy carbon crucible. In a pure sodium trimetaphosphate melt (high Lux acidity), cyclic voltammetry reveals the presence of a diffusion-controlled reduction event at 2.4 V vs Na/Na^+^ (Figure 1C, black trace), proposed to arise from phosphate reduction. Electrolysis at a slightly more negative potential of 2.1 V vs Na/Na^+^ results in collection of pale-yellow pyrophoric crystals in a downstream cold trap, which were spectroscopically confirmed to be pure P_4_ (Figure 1B). The authors estimate an impressive 95% Faradaic efficiency for the 5e^–^ reduction of trimetaphosphate to P_4_.

**Figure 1:**
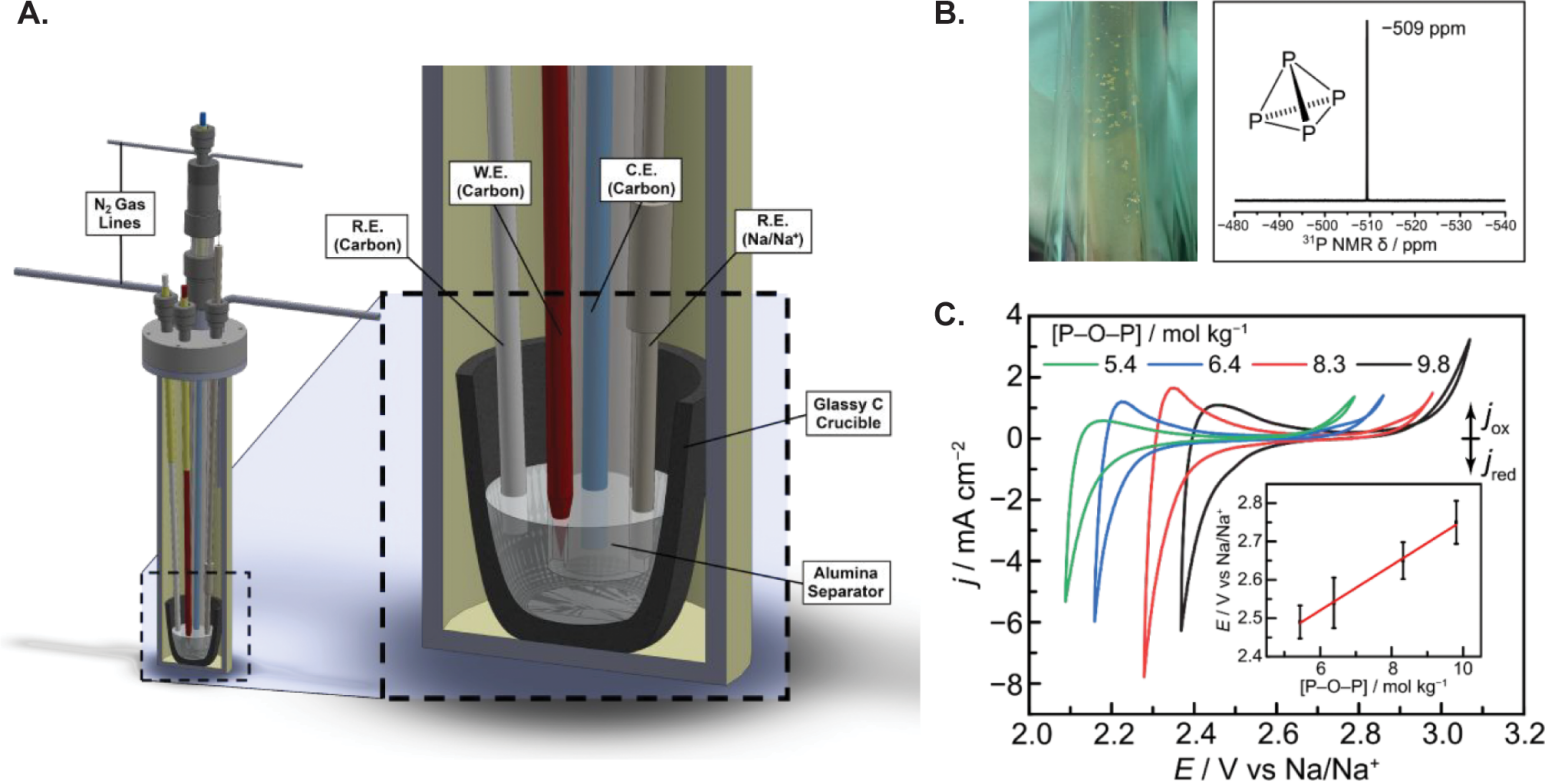
(A) Schematic of the high-temperature electrochemical cell fabricated for this study. (B) Photograph of pale-yellow crystals captured in the cold trap postelectrolysis (left) and corresponding ^31^P NMR spectrum in CS_2_, confirming the product is P_4_ (right). (C) Cyclic voltammograms and OCP measurements (inset) showing that phosphate reduction depends on the electrolyte concentration of phosphoryl anhydrides. Reproduced with permission from ref (1). Copyright 2023. The Authors. Published by American Chemical Society.

Building on this success, the authors investigated additional details of the phosphate reduction reaction as a function of the Lux acidity of the electrolyte. The onset of phosphate reduction shifts to progressively more negative potentials as the Lux acidity decreases (Figure 1C, black to green traces). Furthermore, measurements of open-circuit potential (OCP) under each of these conditions show a linear dependence on the concentration of phosphoryl anhydrides available to act as oxide scavengers (Figure 1C inset). These results confirm that the thermodynamics for P–O cleavage become more favorable as Lux acidity increases, just as the authors originally hypothesized. These efforts parallel another recent advance in sustainable electrosynthesis of silicon nanowires from molten CaSiO_3_.^8^

## References

[ref1] Melville J. L.; A; Surendranath Y. Electrolytic Synthesis of White Phosphorus is Promoted in Oxide-Deficient Molten Salts. ACS Cent. Sci. 2023, in press. 10.1021/acscentsci.2c01336 PMC10037495 36968533

[ref2] Jupp A. R.; Beijer S.; Narain G. C.; Schipper W.; Slootweg J. C. Phosphorus Recovery and Recycling – Closing the Loop. Chem. Soc. Rev. 2021, 50 (1), 87–101. 10.1039/D0CS01150A. 33210686

[ref3] Gilmour R. Phosphorus. In Kirk-Othmer Encyclopedia of Chemical Technology; John Wiley & Sons, 2019. 10.1002/0471238961.1608151902182113.a01.pub3.

[ref4] Geeson M. B.; Cummins C. C. Phosphoric Acid as a Precursor to Chemicals Traditionally Synthesized from White Phosphorus. Science 2018, 359 (6382), 1383–1385. 10.1126/science.aar6620. 29439027

[ref5] Geeson M. B.; Cummins C. C. Let’s Make White Phosphorus Obsolete. ACS Central Science 2020, 6 (6), 848–860. 10.1021/acscentsci.0c00332. 32607432 PMC7318074

[ref6] Ung S. P. M.; Li C.-J. From Rocks to Bioactive Compounds: a Journey Through the Global P(V) Organophosphorus Industry and its Sustainability. RSC Sustain. 2023, 1, 11–37. 10.1039/D2SU00015F.

[ref7] Yang X.; Nohira T. A New Concept for Producing White Phosphorus: Electrolysis of Dissolved Phosphate in Molten Chloride. ACS Sustain. Chem. & Eng. 2020, 8 (36), 13784–13792. 10.1021/acssuschemeng.0c04796.

[ref8] Dong Y.; Slade T.; Stolt M. J.; Li L.; Girard S. N.; Mai L.; Jin S. Low-Temperature Molten-Salt Production of Silicon Nanowires by the Electrochemical Reduction of CaSiO_3_. Angew. Chem., Int. Ed. 2017, 56 (46), 14453–14457. 10.1002/anie.201707064. 28952181

